# Exploring the levodopa-paradox of freezing of gait in dopaminergic medication-naïve Parkinson’s disease populations

**DOI:** 10.1038/s41531-023-00575-0

**Published:** 2023-09-09

**Authors:** Jamie A. F. Jansen, Tamine T. C. Capato, Sirwan K. L. Darweesh, Egberto R. Barbosa, Rogier Donders, Bastiaan R. Bloem, Jorik Nonnekes

**Affiliations:** 1 Radboud University Medical Center, Donders Institute for Brain, Cognition and Behavior, Department of Rehabilitation, Center of Expertise for Parkinson & Movement Disorders, Nijmegen, The Netherlands; 2 University of São Paulo, Department of Neurology, Movement Disorders Center, São Paulo, Brazil; 3 Radboud University Medical Centre, Donders Institute for Brain, Cognition and Behaviour, Department of Neurology, Centre of Expertise for Parkinson & Movement Disorders, Nijmegen, The Netherlands; 4 Radboud University Medical Center, Radboud Institute for Health Sciences, Nijmegen, The Netherlands; 5 Department of Rehabilitation, Sint Maartenskliniek, Ubbergen, The Netherlands

**Keywords:** Parkinson's disease, Parkinson's disease

## Abstract

The relationship between dopaminergic treatment and freezing of gait (FOG) in Parkinson’s disease (PD) is complex: levodopa is the most effective symptomatic treatment for FOG, but long-term pulsatile levodopa treatment has also been linked to an increase in the occurrence of FOG. This concept, however, continues to be debated. Here, we compared the occurrence of FOG between a levodopa-naive PD cohort and a levodopa-treated cohort. Forty-nine treatment-naive patients and 150 levodopa-treated patients were included. The time since first motor symptoms was at least 5 years. Disease severity was assessed using the MDS-UPDRS part III. Occurrence of FOG was assessed subjectively (new freezing-of-gait-questionnaire) and objectively (rapid turns test and Timed Up-and-Go test). The presence of FOG was compared between the levodopa-treated and levodopa-naive groups using a chi-square test of homogeneity. We also performed a binomial Firth logistic regression with disease duration, disease severity, country of inclusion, location of measurement, and executive function as covariates. Subjective FOG was more common in the levodopa-treated cohort (*n* = 41, 27%) compared to the levodopa-naive cohort (*n* = 2, 4%, *p* < 0.001). The association between FOG and levodopa treatment remained after adjustment for covariates (OR = 6.04, 95%Cl [1.60, 33.44], *p* = 0.006). Objectively verified FOG was more common in the levodopa-treated cohort (*n* = 21, 14%) compared to the levodopa-naive cohort (*n* = 1, 2%, *p* = 0.02). We found an association between long-term pulsatile levodopa treatment and an increased occurrence of FOG. Future studies should further explore the role of nonphysiological stimulation of dopamine receptors in generating FOG, as a basis for possible prevention studies.

## Introduction

Freezing of gait (FOG) is among the most mysterious and dramatic symptoms in people with Parkinson’s disease (PD)^1–3^. FOG is defined as a ‘brief, episodic absence or marked reduction of forward progression of the feet despite the intention to walk’^4^. During an episode of FOG, patients have the feeling that their feet are suddenly being glued to the floor^5^. The underlying pathophysiology is not completely unraveled^6,7^, but FOG is associated with disease duration, disease severity, and executive function^8–10^.

Levodopa is the current ‘gold standard’ for the symptomatic treatment of PD^11–13^. An increase in levodopa dosage is also the first step in the management of FOG^3^. The relationship between levodopa and FOG is, however, complex. Shortly after the introduction of levodopa in the sixties of the previous century, two publications suggested that long-term levodopa treatment resulted in an increase in the occurrence of FOG^14,15^. This finding is supported by an analysis of historical textbooks and films—which suggested that FOG was distinctly rare prior to the introduction of levodopa^16^—and by observations on patients with MPTP-induced parkinsonism, who in their untreated phase did not manifest any FOG despite having severe end-stage parkinsonism, although FOG did develop later on following initiation of levodopa^17^. We have referred to this dual effect as the so-called levodopa paradox^18^, but this concept continues to be debated^19,20^.

Controlled studies could shed further light on this apparent levodopa paradox, but controlled experimentation is difficult as it would be unethical to withhold levodopa from persons with PD for several years in healthcare settings where levodopa is available. Therefore, other approaches are needed. In several parts of the world, persons with PD are underserved: levodopa is not available, too expensive, or treatment is delayed because it is difficult to consult a neurologist^21–24^. Such underserved populations (in effect creating levodopa-naive cohorts) provide a unique opportunity to study the levodopa paradox. Here, we compared the occurrence of FOG in a levodopa-naive cohort in Brazil to a levodopa-treated cohort (consisting of persons with PD from Brazil and the Netherlands). We hypothesized that FOG would be more common in persons with PD receiving chronic pulsatile levodopa treatment compared to levodopa-naive PD patients.

## Results

We were able to include 150 levodopa-treated patients (75 from the Netherlands and 75 from Brazil) and 49 Brazilian levodopa-naive patients (Table 1). In the levodopa-treated group, 76 patients (51%) were exposed to other forms of dopaminergic treatment on top of levodopa treatment. Time since onset of motor symptoms and time since diagnosis was longer in the levodopa-treated group compared to the levodopa-naive group (1.2 years difference in time since onset of motor symptoms and 2.1 years difference in time since diagnosis). In addition, disease severity was higher in the levodopa-treated group compared to the levodopa-naive group (7 points on the MDS-UPDRS part III score). No differences between groups were found in mediolateral balance capacity (as assessed with tandem gait) and postural instability (as assessed with the retropulsion test). There were no missing data.

**Table 1:** Study population characteristics.

	Levodopa-naive (Brazil) *n* = 49	Levodopa+ *n* = 150	Levodopa+ (Netherlands) *n* = 75	Levodopa+ (Brazil) *n* = 75
Age (years)	65 [42–90]	66 [33–93]	69 [51–93]	64 [33–91]
Sex (M/F)	30/19	107/43	58/17	49/26
Time since onset of motor symptoms (years)	6.5* [5–20]	7.7* [5–22]	7.5 [5–16]	7.8 [5–22]
Time since diagnosis (years)	4.0* [0.1–9]	6.1* [2–11]	6.1 [2–11]	6.1 [5–11]
Duration levodopa treatment (years)	N/A	5.6 [2–10]	5.6 [2–10]	5.6 [3–10]
Duration dopaminergic treatment (years)	N/A	5.9 [2–10]	5.8 [2–10]	5.9 [3–10]
Exposed to dopamine agonists (*n*, %)	N/A	76 [51%]	26 [35%]	50 [67%]
Levodopa equivalent dosage (g)	N/A	0.79 [0.1–2.1]	0.74 [0.1–1.1]	0.84 [0.2–2.1]
Cumulative lifetime levodopa exposure (g)	N/A	727 [128–4122]	888 [165–2732]	567 [128–4122]
MDS-UPDRS part III [0–136]	30* [12–58]	37* [12–79]	38 [12–79]	36 [12–66]
Frontal Assessment Battery [0–18]	15 [9–18]	15 [9–18]	16 [9–18]	15 [8–18]
Home assessment (*n*, %)	12* [24%]	81* [54%]	75 [100%]	6 [8%]

Values are represented as mean ± range, unless otherwise specified.

* Shows statistically significant difference between the levodopa-naive and the combined levodopa-treated group.

**Fig. 1:**
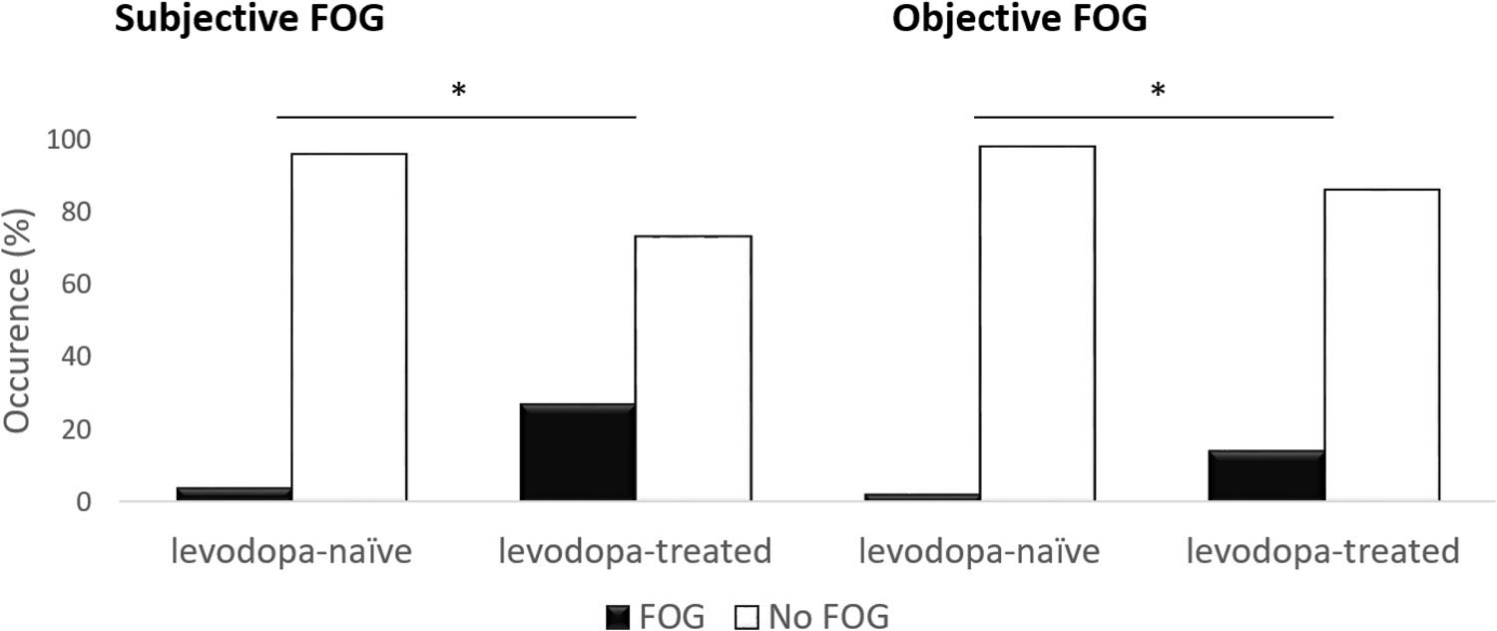
Occurrence of subjective and objective FOG in the levodopa-naive and levodopa-treated cohorts.

Subjective FOG was more commonly present in the levodopa-treated group (*n* = 41, 27%) compared to the levodopa-naive group (*n* = 2, 4%, *X*^2^ (1, *N* = 199) = 11.788, *p* < 0.001, Fig. 1). Chronic levodopa-treatment was associated with subjective FOG (OR = 8.38, 95%CI [2.45, 43.85], *p* = <0.001) in a model which included country of inclusion, assessment location, and FAB-score as covariates. After additional adjustment for disease duration and disease severity, the odds ratio remained virtually unchanged (OR = 6.04, 95%Cl [1.60, 33.44], *p* = 0.006).

Objective FOG was also more commonly observed in the levodopa-treated group (*n* = 21, 14%) compared to the levodopa-naive group (*n* = 1, 2%, *X*^2^ (1, *N* = 199) = 5.372, *p* = 0.02, Fig. 1). In the multivariate analysis, the presence of levodopa treatment was not associated with objective FOG (OR = 2.77, 95%Cl [0.57, 26.87], *p* = 0.226); objective FOG was associated and more likely with higher disease severity (OR = 1.07, 95%Cl [1.03 1.12], *p* < 0.001) and longer time since onset motor symptoms (OR = 1.20, 95%Cl [1.03, 1.41], *p* = 0.018). Country of inclusion, assessment location, and FAB-score were also not associated with the presence of objective FOG.

All objective freezers reported subjective FOG. Only FOG with alternating trembling of the legs or short shuffling steps was seen, and no freezing of the akinetic subtype was observed.

## Discussion

We conducted an observational cohort study in South America and Europe, aiming to explore the levodopa-paradox that has been claimed to be associated with FOG. Specifically, the occurrence of subjective and objective FOG was compared between a treatment-naive cohort (without proceeding exposure to levodopa or any other type of symptomatic medication) and a levodopa-treated cohort. Subjective FOG was significantly more common in the levodopa-treated cohort compared to the levodopa-naive cohort, and this difference remained when potential confounders were taken into account. Objective FOG was also more common in the levodopa-treated cohort compared to the levodopa-naive group, but this difference was no longer significant when confounders were included in the analysis. We only observed FOG with alternating trembling of the legs or short shuffling steps, both in treated and untreated individuals.

This is the first study that assessed the occurrence of both subjective and objective FOG in a cohort that had not received any form of dopaminergic medication in the past. A previous study assessed the occurrence of subjective FOG in an untreated cohort of 30 people with PD in Sub-Saharan Africa^25^. In this cohort, the time since the onset of the first motor symptoms was 7.1 years, and the average UPDRS part III score 41.9 (so ~48.9 when converted to MDS-UPDRS part III score)^26^. Five patients (16.7%) reported subjective FOG, as assessed using the UPDRS part II item 14. This is higher than the 4% of subjective FOG found in our levodopa-naive group, and this difference is unlikely to be fully explained by a greater disease severity in the sub-Saharan cohort (48.9 compared to 41.9 in our Brazilian levodopa-naive cohort). Interestingly, the percentage of subjective FOG found in our levodopa-treated cohort (27%) was also lower compared to previous reports of subjective FOG in patients who were likely treated with dopaminergic medication. In a recent meta-analysis, the prevalence of subjective FOG in patients with a disease duration between 5 and 9 years was 48.4%^27^, which is again somewhat higher compared to our treated cohort. Perhaps differences in ascertainment could explain this discrepancy, as the specific way in which the question about freezing is clarified further to the patient may have an impact on the answers that are being obtained. In line with the literature, we found a lower percentage of objective FOG compared to subjective FOG; this is readily explained by the fact that FOG is notoriously difficult to provoke in a clinical or research setting^28,29^.

There are several complementary explanations for the higher occurrence of FOG in the levodopa-treated group compared to the levodopa-naive group. The first is that the results are in line with the levodopa paradox, suggesting that long-term nonphysiological stimulation of dopaminergic receptors may contribute to the occurrence of FOG^18^. Importantly, at the same time, our data do also support the notion proposed by others that FOG may occur without any previous dopaminergic treatment^16,19,20^, but our findings do suggest that long-term levodopa treatment further increases the risk of developing FOG^18^. This is in line with reports on a new gait phenomenon (which we now call FOG) that appeared to emerge for the first time after the introduction of levodopa in the sixties of the previous century^14,15^. The second explanation is potential confounding by disease duration^8,30–32^, disease severity^30^, or cognitive impairments^10^, but after statistical correction for this, subjective FOG remained significantly more common in the levodopa-treated cohort compared to the levodopa-naive cohort. The results were more convincing for subjective FOG compared to objective FOG (in which treatment effect disappeared after correction for the confounders), possibly because of the much lower numbers of persons manifesting objective FOG compared to subjective FOG (caused by the fact that FOG is notoriously difficult to elicit with an examiner being present).

The question remains what specific further evidence is needed to establish causality between long-term pulsatile levodopa treatment and the occurrence of FOG. An RCT is likely not feasible, as it is unethical to withhold levodopa from persons with PD for several years. The parallel between smoking and lung cancer might be of help here; causality is now generally accepted, despite the lack of RCTs. Hence, capitalizing on the work in this cancer field, replication of our findings in well-designed observational studies, and ideally confirmation in animal models may be of help^33^.

Our study also sheds light on how long-term levodopa treatment may influence the phenotype of FOG, expanding on previous studies in this field^18^. Typically, FOG can be divided into three phenotypes^34^. The first two phenotypes involve freezing with small, forward shuffling steps, and freezing with alternating leg trembling at 3 to 8 Hz. The third, and least common, phenotype is akinetic FOG, during which no movements are observed. It was hypothesized that FOG of the akinetic phenotype would be more common in levodopa-naive patients, while FOG with small, forward shuffling steps or alternating leg trembling would be more common after long-term levodopa treatment^16^. In our study, the numbers of objective FOG in the levodopa-naive group were too small to draw conclusions, but the fact that FOG with alternating leg trembling was observed in a levodopa-naive patient, while no akinetic FOG was seen, argues against levodopa treatment influencing the freezing phenotype.

Although this is the largest cohort assessing FOG in levodopa-naive patients so far, we did not reach the intended sample of 75 levodopa-naive patients, which resulted in less statistical power to examine our hypothesis. This may have impacted the outcome of the multivariate analysis when looking at objective FOG. The COVID-19 pandemic hit Brazil hard and made recruitment of participants in this study very challenging. Moreover, the COVID-19 pandemic also changed clinical practice in Brazil: it accelerated the use of telemedicine^35,36^, thereby increasing the accessibility to specialized care and reducing the number of levodopa-naive patients. A second limitation is that we were unable to correct for possible socioeconomic differences, such as access to nutrition, education, and healthcare, which are obviously present between the Netherlands and Brazil. One may argue that it might have been better to fully perform the study in Brazil, but socioeconomic differences are also large within this country, and likely exist between Brazilian levodopa-naive and levodopa-treated patients. Another limitation of this study is the subjective account of symptom duration. As this is not a longitudinal study, the time since onset motor symptoms was subjectively reported by the participants, and one may question the accuracy. However, there is no reason to assume that the accuracy of this estimate differed systematically between the cohorts.

Our findings set the agenda for further studies exploring the role of nonphysiological stimulation of dopamine receptors in generating FOG, with the ultimate aim to develop improved treatment strategies that carry a lower risk of causing FOG. A much-needed next step would be to explore whether the risk is indeed associated with the nonphysiological pulsatility of the levodopa treatment (e.g., by comparing cohorts receiving immediate or sustained release preparations).

## Methods

### Study overview and selection criteria

An observational cohort study was conducted in the Netherlands and Brazil from January 2019 up to November 2022. We aimed to include two cohorts: 150 people with PD receiving levodopa treatment (75 recruited in the Netherlands and 75 recruited in Brazil); and 75 Brazilian people with PD who had not been treated with any form of dopaminergic medication or natural dopamine supplements (e.g., macuna pruriens). A diagnosis of PD had to be established by a movement disorders expert, according to established international criteria. We only included patients in whom the onset of motor symptoms was at least 5 years ago, as FOG is rare in the early stages of PD^8^. In addition, we only included patients who were able to walk unaided. Participants were excluded when co-morbidity hampered ambulation. In the levodopa-treated group, people were not allowed to receive advanced treatments (deep brain stimulation, subcutaneous or duodenal dopaminergic medication). Levodopa-treated individuals were allowed to be or have been treated with other types of symptomatic dopaminergic medication as well.

### Recruitment

In the Netherlands, participants were recruited via the PRIME-NL cohort^37^. In Brazil, participants were recruited via the coordinating University of São Paulo, and via outpatient clinics of regional hospitals in 16 districts (São Paulo, Rio de Janeiro, Minas Gerais, Piauí, Distrito Federal, Mato Grosso, Bahia, Rio Grande do Norte, Pernambuco, Amazonas, Maranhão, Espírito Santo, Alagoas, Paraná, Santa Catarina, and Rio Grande do Sul). During recruitment, assessors were blinded for the presence of FOG, to prevent selection bias, and patients were unaware of the hypothesis of this study. Measurements took place either at the outpatient clinic or at the participant’s home. The study was approved by the Medical Ethical Committee Arnhem-Nijmegen (NL72917.091.20) and the Ethical Committee of University of São Paulo Medicine School (CEP: 4.707.356), and all participants provided written consent.

### Assessment of patient characteristics

Participants in the levodopa-treated cohort were assessed in the OFF-state at least 12 h after the last intake of dopaminergic medication. We chose to examine patients in the dopaminergic OFF-state, as FOG is notoriously difficult to provoke in the research setting, and is most commonly seen in the OFF-state^34^. For all participants, we collected information on disease duration (i.e., time since motor symptom onset and diagnosis), time since dopaminergic treatment (i.e., levodopa and any other form of dopaminergic medication), levodopa equivalent daily dosage, and cumulative lifetime levodopa exposure^38^. Executive function was mapped using the Frontal Assessment Battery (FAB)^39^, and disease severity was assessed with the MDS-UPDRS part III^40^.

### Assessment of freezing of gait

Subjective presence and severity of FOG were assessed using the New Freezing of Gait Questionnaire (NFOG-Q)^41^. In addition, the presence of FOG was objectively verified by two experienced raters (JJ and JN) using the Timed Up-and-Go test (TUG)^42^ and four rapid 360-degrees turns in alternating directions^43^. Mediolateral balance was assessed using the tandem gait test. For this test, patients were instructed to take 10 steps along an imaginary straight line with their eyes open and their leading foot’s heel against the toe of the other foot^44^. The retropulsion test (as part of the MDS-UPDRS part III) evaluated the presence and quality of balance-correcting steps. Rapid turning and the TUG were videotaped and rated for the presence and phenotype of FOG. Tandem gait and the retropulsion test were also videotaped.

### Statistical analysis

Patient characteristics and postural instability (retropulsion test scores) were tested for normality using the Shapiro–Wilk test and then compared with either an independent samples T-test, or a non-parametric independent samples Kruskal–Wallis test. To identify differences in tandem gait performance and assessment location (at home or at the outpatient clinic), a 2 × 2 chi-square test was performed.

The presence of objective and subjective FOG in the two groups was first univariately compared using a 2 × 2 Chi-square test of homogeneity. We then performed a multivariate analysis involving a binomial Firth logistic regression with levodopa-treatment (yes/no) as categorical variable, with country of inclusion, assessment location, disease severity (MDS-UPDRS part III score), time since onset of motor symptoms and cognitive impairments (FAB scores) as covariates. These covariates were selected because prior studies suggest that they may be associated with both the determinant (longstanding levodopa use) and outcome (freezing of gait) of this study. We separately ran the model without the covariates disease duration and disease severity.

### Reporting summary

Further information on research design is available in the Nature Research Reporting Summary linked to this article.

### Supplementary information

Reporting Summary

## Data Availability

Data are available upon request to the corresponding author.
